# Combined Clear Cell Renal Cell Carcinoma and Chromophobe Renal Cell Carcinoma: A Case Report

**DOI:** 10.7759/cureus.61940

**Published:** 2024-06-08

**Authors:** Abdelrazak Meliti, Hosam Alardati, Manal Khayat, Abdullah Alruqi

**Affiliations:** 1 Department of Pathology and Laboratory Medicine, King Faisal Specialist Hospital and Research Centre, Jeddah, SAU; 2 Department of Pathology and Laboratory Medicine, King Abdulaziz Medical City, Jeddah, SAU; 3 Department of Pathology and Laboratory Medicine, King Fahad Armed Forces Hospital, Jeddah, SAU

**Keywords:** computed tomography, kidney, nephrectomy, multiple tumors, renal cell carcinoma

## Abstract

Renal cell carcinoma (RCC) is a diverse array of cancers arising from renal tubular epithelial cells. RCC presenting with distinct morphological subtypes, such as the simultaneous presence of chromophobe RCC (chRCC) and clear cell RCC (ccRCC) lesions within the same kidney, is rare. We present the case of a 79-year-old female with a history of breast cancer who presented to our facility with right flank pain. Further investigations using CT of the abdomen and pelvis revealed a Bosniak type 4 cyst with a mural nodule in the right kidney. Furthermore, another well-defined, solid lesion measuring 2.8 × 2.6 cm was observed in the same area. The patient underwent a right radical nephrectomy. The macroscopic examination of the kidney revealed the presence of three cysts, with the largest measuring up to 7.5 cm. Moreover, a distinctly demarcated, golden-yellow, solid mass was discerned in the superior pole of the kidney. The mass showed a heterogeneous cut surface with solid and cystic components, measuring 2.8 × 2.6 × 2.0 cm. A less extensive but well-defined, uniform tan mass was also identified within the wall of the largest cyst, which measured 1.2 × 1.0 × 0.7 cm. At this point, the diagnosis of ccRCC and chRCC was established.

## Introduction

Clear cell renal cell carcinoma (ccRCC) is the predominant form of kidney cancer and comprises around 80% of renal carcinomas [1,2]. In the United States, it is the sixth most common cancer in men and the ninth most common cancer in women [3]. The median age of diagnosis of ccRCC is 64 years. The incidence of this condition has been on the rise due to the increased utilization of ultrasonography and CT scans for evaluating patients with varying clinical presentations [4,5]. Bilateral tumors, either synchronous or asynchronous, have been reported in 2-4% of sporadic cases [6]. The ccRCC is responsible for 60-75% of unilateral RCC cases [6]. Despite the majority of ccRCC patients being cured through surgery, metastatic ccRCC accounts for about 20% of newly diagnosed cases [5]. Chromophobe RCC (chRCC) is a rare type of kidney cancer that originates in the cells lining the minor tubules responsible for urine filtration [7]. The incidence of chRCC constitutes 5% of RCC cases and is the third most prevalent RCC subtype after ccRCC and papillary RCC. It can be identified serendipitously or may present clinically [8,9]. According to the literature, chRCC is considered to be a neoplasm of low malignancy. Reported survival rates at five-year and 10-year intervals range from 78% to 100% and 80% to 90%, respectively [10]. Currently, the observed reduction in RCC incidence in developed nations could be partly ascribed to modifications in lifestyle, notably the reduction in smoking rates and the adoption of a healthier lifestyle [11]. We report a case of a morphologically unusual combination of RCC morphological subtypes within the same kidney, with one lesion showing chRCC and another lesion showing ccRCC.

## Case presentation

We present the case of a 79-year-old woman who presented with right flank pain to our hospital on November 1, 2022. Her prior history revealed that she had left breast invasive lobular carcinoma and was undergoing hormonal therapy. A CT scan of the abdomen and pelvis using IV contrast revealed the presence of several renal cysts of varying sizes within the right kidney. One of the cysts showed a mural nodule measuring 1.2 cm in size, which was classified as Bosniak type 4, indicating a high likelihood of malignancy. Another well-defined, solid, and contrast-enhancing lesion measuring 2.8 × 2.6 cm was also observed. The left kidney was normal in size and position. The patient subsequently underwent a right radical nephrectomy. Upon macroscopic analysis, the kidney displayed the presence of three cysts, with the largest measuring up to 7.5 cm in its maximum dimension. Additionally, a well-defined, golden-yellow, cortical, firm mass was observed in the upper pole of the kidney. The mass exhibited a variegated appearance with both solid and cystic cut surfaces and measured 2.8 × 2.6 × 2.0 cm. A smaller, well-circumscribed, tan, firm mass with homogenous cut surfaces was located in the wall of the largest cyst, measuring 1.2 × 1.0 × 0.7 cm on the midportion.

**Figure 1:**
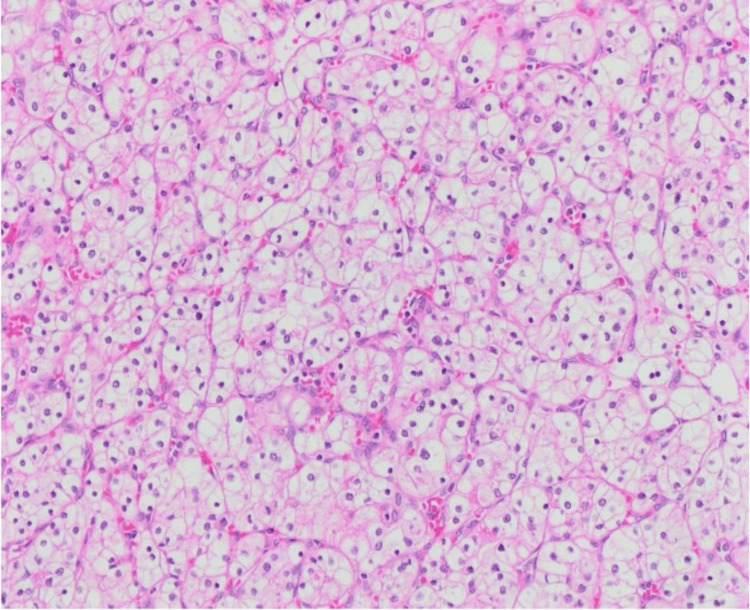
H&E staining reveals a cell exhibiting clear cytoplasm arranged in a nested architecture, accompanied by fine arborizing vascularity (10× magnification)

**Figure 2:**
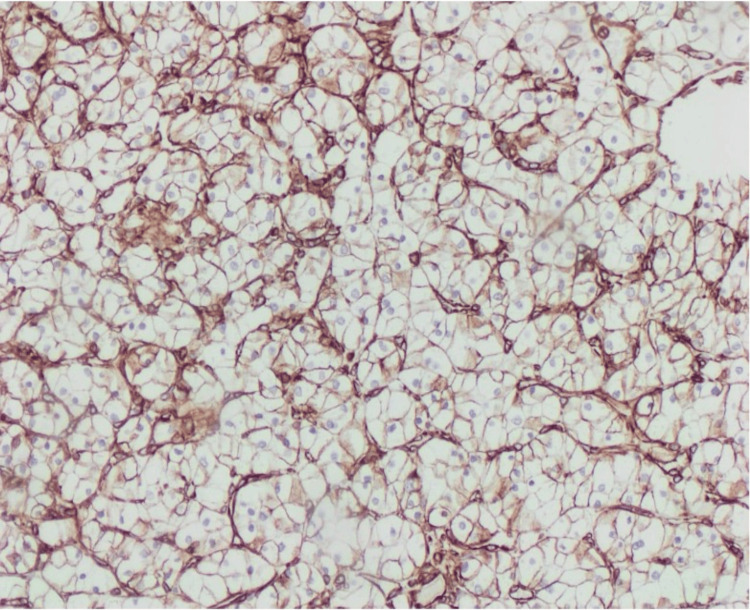
Immunohistochemical staining demonstrates strong, diffuse positivity for vimentin in both the cytoplasm and membrane (10× magnification)

**Figure 3:**
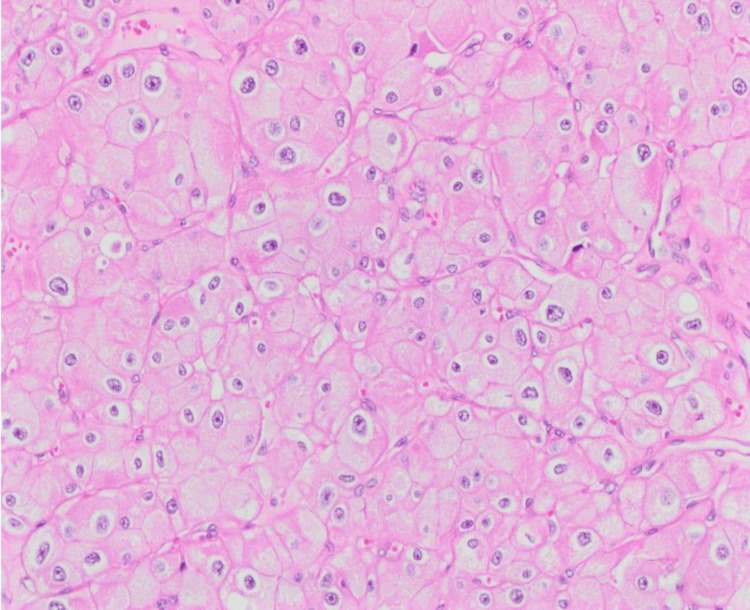
H&E staining reveals tumor cells arranged in a solid nest pattern with reticulated and eosinophilic cytoplasm, accompanied by hyperchromatic wrinkled nuclei (10× magnification)

**Figure 4:**
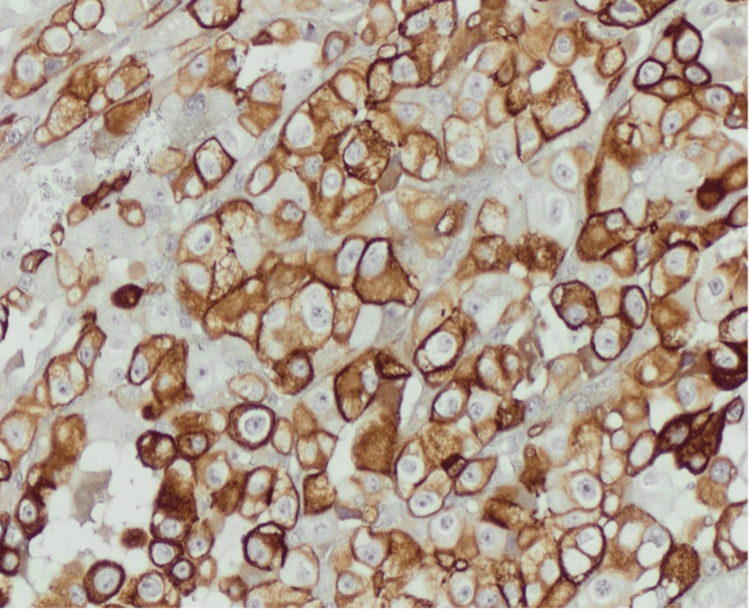
Immunohistochemical staining shows strong, diffuse positivity for cytokeratin 7 in both the cytoplasm and membrane (10× magnification)

Histopathological examination revealed a simple cyst lined by a single layer of flattened epithelium. The larger mass revealed neoplastic growth composed of cells arranged in compact nests surrounded by a network of arborizing small, thin-walled vessels. The neoplastic cells had clear cytoplasm, a distinct membrane, and absent or inconspicuous nucleoli at 400× magnification (Figure 1). Immunohistochemically, the tumor cells showed strong diffuse positive cytoplasmic and membranous staining for vimentin (Figure 2) and were diagnosed as ccRCC, WHO/ISUP grade 1. The smaller mass revealed neoplastic solid growth with nests and sheets separated by thin fibrous septa composed of large polygonal cells with abundant reticular cytoplasm and smaller cells with finely granular eosinophilic cytoplasm. The nuclei were hyperchromatic with irregular, wrinkled nuclear membranes with perinuclear halos. Some cells have binucleation or multinucleation (Figure 3). By immunohistochemistry, the tumor cells showed strong and diffuse cytoplasmic membrane positivity for cytokeratin 7 (Figure 4) and were diagnosed as chRCC. The pathological stage classification (pathological tumor-node-metastasis, American Joint Committee on Cancer, Eighth Edition) for each tumor was pT1a.

## Discussion

The synchronous occurrence of renal tumors exhibiting varied histological subtypes is infrequent and rarely described in the literature [12,13]. We present a case of an unusual event in RCC: chRCC and ccRCC coexisted. Our patient initially presented with complaints of pain in the right flank region. Previously, the diagnosis of RCC relied on presenting symptoms such as flank pain, hematuria, and palpable abdominal masses. However, with the widespread use of noninvasive radiological techniques such as ultrasonography and abdominal CT imaging, most diagnoses are incidental findings [14]. In this regard, CT imaging has emerged as a preferable option for the diagnosis of RCC [15,16]. It contributes significantly to clinical decision-making and is the primary basis for staging and treatment response assessment [17]. The foremost approach for detecting RCC involves imaging techniques depicting diverse radiographic manifestations such as outward growth and nonuniformity attributable to necrotic or hemorrhagic intratumoral regions [18]. Staging of RCC involves assessing the tumor size, extent of invasion outside of the kidney, lymph node involvement, and whether the tumor has metastasized [14]. CT scan imaging with contrast enhancement of the chest, abdominal cavity, and pelvis is required for optimal staging. MRI provides additional information to determine whether the tumor extends into the vasculature [14,19]. In the current case, we utilized a CT scan of the abdomen and pelvis using IV contrast to reveal the presence of several renal cysts of varying sizes within the right kidney. Like the present case, Yang et al. described a synchronous bilateral chRCC with two histological variants, accompanied by a clear cell carcinoma and a cyst [20]. They utilized a contrast CT/MRI scan that showed a solid mass in the right kidney with well-defined margins and exophytic, circumscribed enhancing lesions.

Histopathological diagnosis of malignancy in RCC is typically obtained through renal core biopsy or examination of the partial or radical nephrectomy specimen [21]. A biopsy is recommended prior to ablative therapy or systemic treatment, as appropriate. WHO updated its classification of RCC in 2022, building on previous systems from 2013 and 2016 [22,23]. While most RCCs can be classified based on histological criteria, some tumors display features characteristic of multiple subtypes, posing a diagnostic challenge [24]. However, careful evaluation of cytological features, growth patterns, immunophenotype, and genetic alterations can usually enable a proper diagnosis. In some cases, RCCs cannot be assigned to any specific category and are designated as unclassified RCCs. Recent molecular characterization of aggressive, unclassified RCC revealed distinct subsets with different genetic alterations [25]. Macroscopically, ccRCC tumors are typically golden yellow with hemorrhagic, necrotic, and cystic areas [14]. In the current case, the macroscopic analysis revealed the presence of three cysts, with the largest measuring up to 7.5 cm in its maximum dimension in the kidney. Additionally, a well-defined, golden-yellow, cortical, firm mass was observed in the upper pole of the kidney. Similarly, a case report of coexisting papillary and ccRCC in the same kidney by Ustuner et al. revealed a macroscopic picture that included orange and yellow-colored nodules and small cystic cavities [26]. Further, microscopy of tumors showed solid nests and sheets of carcinoma cells interspersed by a prominent network of delicate blood vessels. Microscopically, ccRCC consists of tumor cells with clear cytoplasm arranged in nests or tubules surrounded by a rich vascular network [27]. A variable proportion of granular eosinophilic cytoplasm may be observed. The Fuhrman grading system is widely used for ccRCC, defining four nuclear grades based on increasing nuclear size, irregularity, and nucleolar prominence. The grading system has prognostic value in ccRCC [28,29]. Alhusban et al. reported a Fuhrman grade 2 tumor that revealed metastases during dissection in one of the 20 para-aortic lymph nodes, while the adrenal gland exhibited focal hyperplasia [30]. The current case report highlights the effectiveness of the utilization of histopathological and molecular characterization for the diagnosis of RCC.

## Conclusions

Our case report highlights the coexistence of ccRCC and chRCC within a single kidney. ccRCC is the most common subtype of RCC and is known to be aggressive and metastasize early. On the other hand, chRCC is less common and generally has a better prognosis. Early detection of associated ccRCC is crucial to prevent early metastasis. Our case emphasizes the importance of careful evaluation and diagnosis of renal tumors, as early detection and treatment can significantly improve patient outcomes. Radical nephrectomy remains the gold standard for treating renal tumors, and close follow-up is recommended for patients with a history of malignancy.
